# Heatwave Events and Mortality Outcomes in Memphis, Tennessee: Testing Effect Modification by Socioeconomic Status and Urbanicity

**DOI:** 10.3390/ijerph16224568

**Published:** 2019-11-18

**Authors:** Ying Li, Cem Akkus, Xinhua Yu, Andrew Joyner, Jennifer Kmet, David Sweat, Chunrong Jia

**Affiliations:** 1 Department of Environmental Health, College of Public Health, East Tennessee State University, Johnson City, TN 37614, USA; liy005@etsu.edu; 2 School of Public Health, University of Memphis, Memphis, TN 38152, USA; cem.akkus@lebonheur.org (C.A.); xyu2@memphis.edu (X.Y.); 3 Children’s Foundation Research Institute, Le Bonheur Children’s Hospital, Memphis, TN 38103, USA; 4 Department of Geosciences, East Tennessee State University, Johnson City, TN 37614, USA; joynert@etsu.edu; 5 Shelby County Health Department, Memphis, TN 38105, USA; jennifer.kmet@shelbycountytn.gov (J.K.); david.sweat@shelbycountytn.gov (D.S.)

**Keywords:** heatwave, mortality, socioeconomic status, urbanicity, relative risk, Memphis

## Abstract

Heatwave studies typically estimate heat-related mortality and morbidity risks at the city level; few have addressed the heterogeneous risks by socioeconomic status (SES) and location within a city. This study aimed to examine the impacts of heatwaves on mortality outcomes in Memphis, Tennessee, a Mid-South metropolitan area top-ranked in morbidity and poverty rates, and to investigate the effects of SES and urbanicity. Mortality data were retrieved from the death records in 2008–2017, and temperature data from the Applied Climate Information System. Heatwave days were defined based on four temperature metrics. Heatwave effects on daily total-cause, cardiovascular, and respiratory mortality were evaluated using Poisson regression, accounting for temporal trends, sociodemographic factors, urbanicity, and air pollution. We found higher cardiovascular mortality risk (cumulative RR (relative risk) = 1.25, 95% CI (confidence interval): 1.01–1.55) in heatwave days defined as those with maximum daily temperature >95th percentile for more than two consecutive days. The effects of heatwaves on mortality did not differ by SES, race, or urbanicity. The findings of this study provided evidence to support future heatwave planning and studies of heatwave and health impacts at a coarser geographic resolution.

## 1. Introduction

The overall rising temperature in the 21st century resulting from climate change is likely to be the most serious global health threat of the century. Heatwaves are natural hazards that pose significant risks to human health [1]. Earlier U.S. studies focusing on large metropolitan areas consistently reported that exposure to high and extreme temperatures in the warm seasons was associated with higher mortality risks [2,3,4,5,6]. Heat-related risks show significant heterogeneity across the U.S. and tend to be higher in northern than southern locations in the nation [7,8,9]. Studies at the state level also found considerable spatial variations in heat-related risks [10,11]. Extreme heat events directly cause deaths, as well as diseases that ultimately lead to deaths, e.g., cardiovascular and respiratory mortality [12,13]. Studies projected that without further reductions in greenhouse gas emissions, the impacts of heatwaves on mortality are likely to be profound in the U.S. later this century [14,15].

Despite the well-documented association between heatwaves and mortality, fewer studies have examined how heat-related mortality risks differ by socioeconomic status (SES) and location within a city or county. Social disadvantages, such as low SES, minority, blighted communities, and social isolation, are likely to increase an individual’s vulnerability; however, previous findings were inconsistent regarding their interactions with heat-related health risks. Several studies demonstrated that heatwaves disproportionally affected mortality among black [16,17,18,19] and low-income residents [20]. Another study examined the interactions of extreme heat with SES and area of green space and reported that the odds of cardiovascular mortality during heatwaves were higher among non-married individuals and those in ZIP codes with more non-green space in eight Michigan cities [11]. On the other hand, a study reported that the elevated risk of dying from cardiovascular diseases did not differ by SES or residence in the northeastern U.S. [13]. In King County, Washington, the risk of dying on a heat day was not altered by individual-level characteristics, such as gender, race, education, marital status, Hispanic origin, or tobacco use [21].

Memphis, Tennessee (TN), is a Mid-South metropolitan area with high morbidity, poverty, and racial segregation rates, serving as a good testbed for investigating heatwave impacts and the sociodemographic modifiers. The city of Memphis is the regional center and seat of Shelby County, TN. Memphis has a high percentage of African Americans (67%) and a poverty rate of 30% among African Americans [22]. The leading causes of death in Shelby County during 2006–2010 were cancer (210 per 100,000), heart disease (178 per 100,000), and stroke (59 per 100,000), all higher than the state (Tennessee) and national rates [23]. Health disparities are distinct in Shelby County; for instance, high mortality rates occur in low-income communities, as illustrated in Figure S1. The climate zone where Memphis is located has hot and humid summers: The average high temperature in July and August is approximately 92 °F, and relative humidity is commonly greater than 65%. Heat indexes frequently exceed 100 in summer, and the National Weather Service-Memphis office often issues heatwave warnings and advisories in the area. A study of the 1980 heatwave in Memphis reported 83 heat-related deaths, most of whom were elderly, poor, black, inner-city residents [16].

A few national and regional heatwave studies have briefly reported mortality risks during extreme heat events in Memphis [7,8,9]. However, the previous analyses were based on outdated, county-level mortality data, and the findings were inconsistent. Also, earlier studies suggested that national heatwave warning systems might not be appropriate for all regions, given the profound differences in climate patterns and demographic composition across the U.S. [23]. Nevertheless, none of the national or regional heatwave studies in Memphis has tested and compared different heatwave metrics. The objective of this study was to examine the effects of heatwave on total and cause-specific mortality in Memphis using the latest mortality data. We were particularly interested in whether the associations between heatwaves and mortality differ by urbanicity and SES of the residents, and which heatwave definition is the most effective in predicting heat-related mortality.

## 2. Methods

### 2.1. Study Period and Population

We focused on the warm season, i.e., May through September, in Shelby County for the latest 10-year period from 2008 to 2017. Elevated mortality risks associated with heatwaves are most likely to occur during these months in the U.S. [8,24]. The total population of Shelby County was relatively stable, increasing slightly from 915,000 in 2008 to 937,000 in 2017 [25].

### 2.2. Data Sources

Meteorological data and heatwave definitions. Daily temperature measurements were used to investigate the association between heatwaves and mortality, as earlier studies generally reported the heatwave effect on health was acute and lasted only for three to four days [8,26]. Temperature data, including daily mean, minimum, and maximum for the study period recorded at the Memphis International Airport Weather Station, were downloaded from the Applied Climate Information System (ACIS, http://www.rcc-acis.org/). Previous studies used various definitions of heatwaves to quantify the health impacts of heatwaves since population acclimatization to heat is diverse across different regions [27]. This study applied four distinct heatwaves (HW) definitions adopted from Kent et al. [24], as listed in Table 1. These definitions used different temperature metrics, including mean, maximum, and minimum air temperature, and included both relative measures (HW1–HW3) and one absolute measure (HW4).

Mortality data. The daily mortality data of 77,662 death records in 2008–2017 were based on Shelby County resident death certificate data from the Tennessee Department of Health and analyzed in collaboration with the Shelby County Health Department epidemiology team. Figure S1 shows the mortality incidence rate by census tract in Shelby County during the study period. After excluding unmatched addresses or those that were geocoded outside of Shelby County, 71,306 records remained for analysis, of which 28,545 occurred from May through September. For each record, we extracted the date of death, the underlying cause of death coded following the International Statistical Classification of Diseases (ICD-10, the 10th revision), rules, age, gender, race, and the address of residence. We focused on four categories of the underlying causes of death: Exposure to excessive nature heat-hyperthermia (ICD-10 code X30), all-cause mortality, diseases of the respiratory system (ICD-10 codes started with the letter J), and diseases of the circulatory system (ICD-10 codes started with the letter I). Earlier studies linked heatwaves with increases in all the four categories of deaths [8,12,24,29].

Heatwave events and mortality are more likely associated among elderly populations, and thus we limited our analyses to the elderly population aged 65 and above. As a result, a total of 17,935 deaths were included, with 7948 (44%) being aged 65–79 and 9987 (56%) aged 80 and above. Daily counts of deaths were computed for each of the four underlying causes of deaths considered in this study.

Covariates of analysis. The covariates and potential confounding and interaction variables included the year, day of the week (DOW), age (65–79 and 80+), sex, race (Black, White, and Other), urbanicity of the residence, poverty level, and air pollution. We classified the residence as urban or rural following the U.S. Census Bureau’s definition of the urban area [30]. We classified the neighborhood of the residence as a high- or low-income area based on the poverty line to represent the SES of the deceased person. The US national average poverty rate was 15.1% in 2010 [31]. We used 150% of the national average rate, i.e., 15.1% × 150% = 22.6%, and rounded it to 20% as the cutoff value to classify a census tract as a high- or low-income area.

Numerous studies have reported elevated mortality resulting from exposure to air pollution [32,33,34], and the mortality effect of air pollution was found to be independent of that of air temperature [35]. Our previous study also addressed the socioeconomic and racial disparities in air pollution exposures in Memphis [36]. The interactive effect of air pollution and temperature on mortality has been particularly strong for ground-level ozone, which peaks during warm months [37]. We thus included ozone levels as a covariate. Daily average ozone concentrations were obtained from the U.S. Environmental Protection Agency’s Air Quality System [38]. We used the monitoring data collected at the Frayser Station during 1 January 2008–9 March 2011 and the National Core Station during 10 March 2011–31 December 2017. The National Core Station was more representative as it was located at the county center but started operations in early 2011.

### 2.3. Statistical Analysis

The dependent variables for all the analyses were daily death counts, which might be stratified by individual demographic factors (age group, race, sex, etc.) and neighborhood characteristics (see below for model-specific dependent variables). The association between daily mortality and heatwave events was examined using Poisson regression models, as deaths are rare events. We started with the following model to estimate the relative risks (RRs) of mortality on heatwave days and 1, 2, and 3 days after the heatwave days (lag1, 2, and 3).

log [E(Y_t_)] = Year + DOW_t_ + HW_t_ (or HW lag1, 2, or 3), (1a)

where E(Y_t_) is the expected mortality on day t assumed to follow an over-dispersed Poisson distribution [5]. HW_t_ = 1 if day t was a heatwave day by a heatwave definition, and 0 otherwise; lag1 = 1 if it was a heatwave day 1 day before, and 0 otherwise; lag2 = 1 if it was a heatwave day 2 days before, and 0 otherwise; lag3 = 1 if it was a heatwave day 3 days before, and 0 otherwise; Year = categorical variable for year; DOW_t_ = categorical variable for day of the week. Model (1a) estimated the county-level RRs, which are adjusted for the overall year trend and short-term DOW effects. Model (1a) fitted with a canonical log link was equivalent to a log-linear model.

In addition, we also estimated the cumulative risks of mortality of HW days using a distributed lag nonlinear model (DLNM) for Model (1) [9,39,40,41].

log [E(Y_t_)] = Year + DOW_t_ + f(cross-basis(HW_t_,lag1, lag2, lag3)), (1b)

In the DLNM model, HW statuses of both current and 1–3 lag days were in the same model, represented by a bi-dimensional cross-basis transformation of both current and lag terms, fitted with a natural cubic spline function to model the possible non-linear association between HW and daily mortality. The DLNM means the current day mortality is predicted by both current and 1–3 lag days, which is equivalent to the idea that the effect of HW on mortality will be distributed over the current day and three follow-up days (forward interpretation in time). The cumulative effect will be roughly the sum of effects from the heatwave day and three subsequent days. Details of DLNM methodologies can be found in references [39,42].

Models (1a and 1b) were run separately for HW days, 3 lag days, and cumulative heatwave and lag days, 4 heatwave definitions, and 3 categories of mortality (all-cause, cardiovascular, and respiratory mortalities). RRs in these different scenarios were generated from a total of 60 models.

We then added an interaction item in Model (1) to evaluate whether heatwave effects differed by sex, age group, poverty level, urbanicity, or race:

log [E(Y_t_)] = Year + DOW_t_ + HW_t_ + modifier + HW_t_×modifier (2)

Model (2) was then run for 4 heatwave definitions and 3 categories of deaths, respectively, yielding a total of 60 models. In this interaction model, we included only the heatwave status of the current day. Separate lag effect interaction models were also explored but not reported in this paper due to non-significant results. The dependent variable, daily death counts, were cross-tabbed by modifiers. For example, for assessing the interaction between HW and sex, Y_t_ was the daily death counts for either males or females separately. If there was no death for males (or females) on a specific day, zero was filled for that day.

We also constructed the following complete model to evaluate the heatwave effects on mortality, accounting for each of the covariates and potential confounders:

log [E(Y_t_)) = Year + DOW_t_ + HW_t_ (or lag1, 2, or 3) + Covariates (Age group, Poverty, Urbanicity, Race, Sex, and Ozone) (3)

Model (3) was repeated for the three types of deaths; for the four HW definitions; and for heatwave days, lag1, lag2, and lag3 days. In this model, daily death counts were cross-tabbed by age group, poverty, urbanicity, race, and sex. Zeros were filled for cells with no death, resulting in a sparse dataset. Model (3) did not include relative humidity (RH) as a covariate because it was a function of temperature, and a recent global study reported that RH did not influence heat-related mortality [43]. We considered Model (3) an overfit model and used it to confirm the results from simple models.

The address geocoding was completed in ArcGIS (Version 10.3.1, Esri Inc., Redlands, CA, USA). All the statistical analyses were performed in SAS (Version 9.4, SAS Institute, Cary, NC, USA), except that the DLNM models were performed using the package “dlnm” [42] in R (Version 3.6.1, www.r-project.org). No multiple comparison adjustment was made, as most of the comparisons are non-significant at the 0.05 level.

## 3. Results

### 3.1. Daily Temperature Descriptive Statistics

Table 2 summarizes the descriptive statistics of daily temperature for the study period. Using daily temperature data and the four heatwave definitions selected for this study (Table 1), the frequencies of heatwave events were assessed for the study period. The four definitions generated the numbers of heatwave days in different durations, ranging from 3.2 heatwave days per year (2% of the warm season) under HW1 to 21.5 days per year (14% of the warm season) under HW4 (Table 1).

### 3.2. Descriptive Statistics of Mortality

#### 3.2.1. Mortality Attributed to Exposure to Excessive Natural Heat (X30)

The ICD-10 code X30 refers to death due to exposure to excessive natural heat, which is the direct heat-related underlying cause of death. There were a total of 17 deaths with the code X30 in Shelby County over the 10-year study period. Seven of those deaths occurred in 2010, five in 2015, and one death per year in 2008, 2009, 2011, and 2017. Except for one event, all events occurred on a day that was classified as a heatwave day by at least one of the definitions (six under HW1, five under HW2, four under HW3, and 16 under HW4). Only three events, all in 2010, occurred on a day that was classified as a heatwave day based on all four definitions. The only event that occurred on a day not considered a heatwave day based on any of the four heatwave definitions was also checked for possible lag effects. Even when a lag1, 2, or 3 was taken into account, none of the lag days included any heatwave classified days based on any of four definitions. Overall, direct heat-related deaths were relatively rare in Memphis during the study period.

#### 3.2.2. All-Cause, Cardiovascular Disease, and Respiratory Mortality

The total number of all-cause deaths of people aged 65 years and above was 17,935 in Memphis over the 10 years. The distribution of deaths across age groups, poverty levels, genders, and races was generally balanced, except that numbers of other races were small (Table 3). The majority of deaths were urban residents, and only 9% of deaths were rural residents. Disease-specific deaths showed patterns very similar to those of all-cause deaths.

The mortality risk varied by different heatwave definitions. It was notable that the HW4 definition captured many more deaths (2350, Table 3) than the other heatwave definitions, as HW4 used a low threshold of 95 °F. Although the numbers of heatwave days were similar between HW2 and HW3, the actual heatwave days under HW2 and HW3 were different. HW3 should be more related to heatwave shock with its use of maximum temperature for a prolonged period in its definition. Although HW2 and HW3 resulted in similar heatwave duration (32 vs. 35 days), HW3 days captured more deaths than HW2, implying higher mortality risk under HW3.

### 3.3. Association between Heatwaves and Mortality

**Figure 1:**
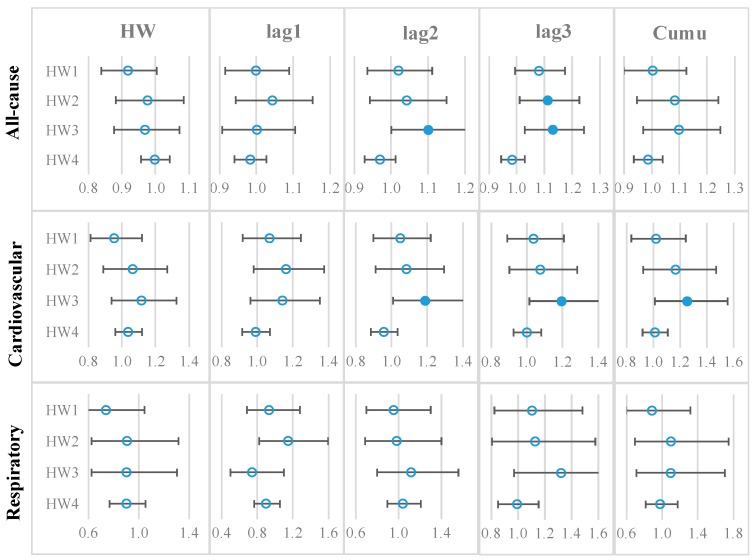
Relative risks (RRs) of all-cause, cardiovascular, and respiratory mortalities on heatwave days and 1–3 lagged days. Note: Each dot indicates the mean RR, and the error bar indicates the 95% confidence interval (CI). A solid dot denotes that a mean RR is greater than 1 and statistically significant, and an empty dot denotes that a mean RR is statistically not different from 1.

Figure 1 summarizes the RRs and 95% confidence intervals (CIs) of all-cause, cardiovascular, and respiratory mortalities on heatwave and 1–3 lagged days, and the cumulative RRs.

For all-cause mortality, there were significant risks on lag3 day based on HW2 (RR = 1.11, 95% CI: 1.01, 1.23) and lag2 day and lag3 day based on HW3 (RR = 1.10 and 1.13, respectively). Based on HW3, cardiovascular mortality increased by 20% on lag2 day (RR = 1.20, 95% CI: 1.01–1.41) and by 20% on lag3 day (RR = 1.20, 95% CI: 1.01–1.41), with an overall increase of 25% (cumulative RR = 1.25, 95% CI: 1.01–1.55) during heatwave days. HW days appeared to have no effects on respiratory mortality (mean RR values < 1).

The results summarized in Figure 1 were confirmed by the full Model (3) that controlled for all potential socioeconomic variables (sex, age, poverty level, and race) and urbanicity levels. Results in Tables S1–S3 confirmed that: (1) heatwave days had no effects on all-cause mortality; (2) heatwave days determined by definitions HW1–3 had increased risks of cardiovascular mortality on lagged 2–3 days; (3) heatwave days showed no effects on respiratory mortality.

Moreover, Model (3) revealed the effects of the time, SES, and urbanicity. For the two temporal variables, the year had significant effects on mortality, indicating the mortality rate changed over the years, but DOW did not show significant effects on mortality. Poverty had significant effects on all-cause mortality (Table S1A–D), but not on cardiovascular mortality (Table S2A–D) or respiratory mortality (Table S3A–D). Residents in urban census tracts had significantly higher mortality risk compared to those in rural tracts for all three mortality outcomes. Daily ozone levels did not affect mortality.

### 3.4. Interactive Effects of Sociodemographic Variables and Urbanicity

We found that there was no interaction between heatwave and sociodemographic variables in predicting daily mortality. As summarized in Table 4, the coefficients of the interaction terms in Model (2) were insignificant for various scenarios, except for the HW4 and race interaction. The results indicated that the effects of heatwaves on mortality were not altered by individual-level characteristics (age, sex, and race) or census-tract-level characteristics (urbanicity and poverty level).

## 4. Discussion

Excess mortality during heatwave events did not appear to be a significant public health issue in Memphis. The 1980 single heatwave event resulted in 83 direct heat-related deaths due to heat exhaustion, heatstroke, or systemic hyperthermia [16]. In contrast, only 17 direct heat-related deaths were recorded in Memphis during our 10-year study period. The 1980 heatwave was a record-breaking extreme heat event, with a maximum temperature higher than 100 °F for 15 consecutive days. During our study period, the longest period with the maximum temperature higher than 100 °F lasted four days, which happened twice in August 2010 and again in June–July 2012. Moreover, the local government implements various heat risk management measures in planning for heatwave events, such as informing the public about heat warnings and opening and widely adverting cooling centers in the city, which may have effectively prevented exposure of the public to excessive heat.

Local heat-health early warning systems should be based on identified local heatwave definitions that are optimal for protecting public health [24,44]. In this study, we tested four distinct heatwave definitions based on different temperature metrics, including mean, maximum, and minimum temperatures. HW4 defines heatwaves as extreme temperatures lasting for one day or longer, but the remaining three definitions require extreme temperatures to continue for at least two days. As a result, HW4 generated a significantly larger number of heatwave days than the other three definitions; however, no mortality increase was found on heatwave days defined by HW4. HW3 (maximum daily temperature) appeared to be the most effective metric in predicting heatwave-related mortality. Based on this finding, we suggest future planning for heatwaves in Memphis be focused on daily maximum temperatures. In contrast, a study in Alabama, another southern state, suggested that mean daily temperature might be the most effective metric for heatwave warning systems [24].

We did not find significant impacts of heatwaves on the overall mortality in Memphis during the study period. An earlier U.S. national study on heat-related mortality risks reported lower risks in the South than those in the Northeast or Midwest and no mortality increase during heatwaves in some southern cities, such as Charlotte, NC, which has a similar warm season climate pattern to Memphis [8]. Our findings generally agreed with that study; however, our analysis of cause-specific mortality showed that under HW3, cardiovascular mortality increased by 25% (cumulative RR = 1.25, 95%CI: 1.01–1.55) during heatwave days. In particular, the 2–3 days lagging effects were more evident. After adjusting for all SES and demographic factors, the effects remained significant (*p* = 0.007 for lag2 and 0.018 for lag3), as shown in Table S3C,D. Heatwave effects on cardiovascular mortality were consistent across the four definitions, although the results based on the other definitions were non-significant. Surprisingly, heatwave days showed no effects on respiratory mortality. Our finding related to respiratory mortality is different from some earlier studies, which generally reported increases in respiratory deaths during heatwaves [27,45,46,47].

Moreover, our individual-level analysis did not find heterogeneity in heat-mortality risks among populations in different races, SES, or residential locations across the county. Notably, income and neighborhood urbanicity levels did not appear to be an effect modifier that exacerbates vulnerability to heatwaves in Shelby County. Studies have documented considerable variation in SES, racial distributions, land use types, and health outcomes by census tract in Memphis [36,48,49], exemplified by Figure S1. However, the crosstab between various SES and urbanicity levels might have created many zeros or small sample size cells, resulting in a diminished power to detect interaction effect.

Previous studies reported positive associations between short-term changes in ozone and mortality [33,50] and its interactive effect with daily temperature [37]. The daily ozone levels remained low in Memphis during warm seasons (mean = 33 ppb), and we found no significant impacts of short-term ozone exposure on mortality. This result is consistent with the findings from a study in Allegheny County, Pennsylvania, which reported no contribution to excess mortality by ozone during a 1988 heatwave [51]. Unfortunately, the lack of monitoring data prevented us from examining other important air pollutants, such as fine particulate matter and nitrogen dioxide.

We acknowledge several limitations of this study. First, the temperature data were obtained from only one single weather station, which might not account for the within-city variations in temperature, especially the differences between urban and rural areas. The urban center may have higher surface temperatures due to the “urban heat island” effect. Further analysis may use high-resolution temperature data to capture the variation in heat exposure. Second, the mortality rate calculations were based on 2010, assuming a stable population size over the entire study period. Nevertheless, this approach was legitimate, given the stable population size in Shelby County. Third, for heatwave definitions 1, 2, and 3, we had small numbers of heatwave days over the study period. The small daily mortality numbers might lead to reduced statistical power when exploring various interactions between heatwave and SES. Fourth, due to the unavailability of individual income data, poverty at the census tract level was used to represent an individual’s income level, which may not capture the variability among individuals. Fifth, we have small death counts for some disease-specific deaths (e.g., respiratory deaths in Table 3), and during cross-tabbing among HW and many modifiers, small death counts and filled zeros in many cells resulted in a sparse dataset. This would lead to reduced statistical power and yield over- or under-estimates of the true HW effect. Sixth, we were mainly interested in investigating what the whole effects of HW were and which definition of heatwaves might be the most effective metric in predicting mortality outcomes and for heatwave warning systems at the study location. As a result, the heatwaves were only modeled as indicators rather than using actual temperature values. As suggested by anonymous reviewers, the right tail (higher temperature) might be more informative than the average temperature in predicting daily mortality. Modeling temperature as a non-parametric smoothing term similar to those in non-linear additive models may reveal interesting findings. In addition, including both the heatwave indicator and continuous temperature in the model will assess the “added” heatwave effect [40]. We will examine the granularity of temperature effects in future research. Finally, analysis within one area may not be adequate for examining heatwave effects due to small spatial variations. We found quite homogeneous heat-related mortality risks in terms of SES and residence. Still, our study well serves as a case study for the impact of heatwaves on mortality in a large metropolitan area in Mid-South U.S. It will also be worthwhile examining heat-related morbidity risks using emergency room visits data for future studies [52,53].

## 5. Conclusions

This study analyzed the association between heatwaves and daily mortality among elderly ages 65 years and above in Shelby County, TN in 2008–2017, and investigated the effects of SES and urbanicity level on heatwave-related mortality risks based on two statistical models, four distinct heatwave definitions, and three categories of mortality outcomes. We found a slight increase of all-cause mortality due to heatwave events, and this was mostly driven by an approximately 25% increase (RR = 1.25, 95% CI: 1.01–1.55) of cardiovascular mortality based on the HW3 definition, i.e., maximum daily temperature >95th percentile for more than two consecutive days. We did not find significant interaction effects between heatwave effects and sociodemographic parameters (age, sex, race, poverty, and urbanicity level of residence) or air pollution. Our findings suggested that the cardiovascular mortality risk due to heatwave events remained nontrivial in the South region of the U.S., despite the high availability of air conditioning and population acclimatization, contributing to the relatively lower vulnerability to heat-related mortality. Future research is needed to extend the analysis to a longer period, validate the generalizability of our findings in other large urban areas, and improve heat exposure assessment at the community and individual levels.

## Figures and Tables

**Table 1:** Heatwave definitions used in this study.

Heatwave Abbreviation	Definition	Reference	Total HW Days
HW1	Minimum daily temperature >95th percentile for ≥2 consecutive days ^1^	Anderson and Bell 2011 [8]	45
HW2	Mean daily temperature >95th percentile for ≥2 consecutive days	Anderson and Bell 2011 [8]	32
HW3	Maximum daily temperature >95th percentile for ≥2 consecutive days	Anderson and Bell 2011 [8]	35
HW4	Maximum daily temperature >35 °C (95 °F) for ≥1 day	Tan et al. 2007 [28]	215

^1^ Percentiles were based on daily temperatures in warm seasons (May–September, 153 days per year) of the 10-year study period.

**Table 2:** Descriptive statistics of daily temperature metrics in May–September during 2008–2017.

Descriptive Statistics	Daily Temperature Metric (°F)		
	Maximum	Minimum	Average
Mean	88.1	69.8	78.9
Maximum	106	84	94.5
Minimum	58	36	47
95th percentile	98	79	88
99th percentile	100	81	90

**Table 3:** Death numbers in May–September and heatwave days due to all causes, cardiovascular diseases, and respiratory diseases in Shelby County in 2008–2017.

	All-Cause Mortality					Cardiovascular Morality					Respiratory Mortality				
	Total	HW1	HW2	HW3	HW4	Total	HW1	HW2	HW3	HW4	Total	HW1	HW2	HW3	HW4
Total	17,935	486	371	400	2530	6081	171	137	150	882	1673	38	34	36	223
Age															
Age 65–79	7948	228	164	181	1116	2446	73	58	57	337	748	16	15	19	109
Age 80+	9987	258	207	219	1414	3635	98	79	93	545	925	22	19	17	114
Poverty															
Low	9072	252	186	214	1280	3192	85	65	81	467	782	21	18	19	96
High	8863	234	185	186	1250	2889	86	72	69	415	891	17	16	17	127
Urbanicity															
Rural	1573	35	28	33	221	493	13	9	8	69	179	2	3	4	29
Urban	16,362	451	343	367	2309	5588	158	128	142	813	1494	36	31	32	194
Gender															
Female	7883	216	211	179	1095	2698	76	60	65	375	775	16	16	18	94
Male	10,051	270	160	221	1435	3382	95	77	85	507	898	22	18	18	129
Race															
Black	10,202	265	200	235	1,454	3359	92	78	88	501	1136	26	20	25	165
White	7529	217	168	162	1055	2645	77	59	60	374	521	11	14	11	54
Other	204	4	3	3	21	77	2	0	2	7	16	1	0	0	4

Note: “HW1, HW2, HW3, and HW4” refer to death numbers on heatwave days determined by definitions 1, 2, 3, and 4, respectively.

**Table 4:** Interactions of heatwave days with age group, income level, urbanicity, sex, and race.

All-Cause	Age	*p*	Poverty	*p*	Urban	*p*	Sex	*p*	Race	*p*
HW1	−0.109	0.249	−0.054	0.562	0.221	0.229	−0.020	0.828	0.109	0.204
HW2	0.003	0.978	0.016	0.879	0.168	0.413	0.034	0.749	0.134	0.169
HW3	−0.040	0.701	−0.122	0.239	0.069	0.715	−0.033	0.750	−0.068	0.473
HW4	0.008	0.858	−0.003	0.950	0.006	0.936	0.032	0.467	−0.018	0.654
Cardiovascular										
HW1	−0.103	0.542	0.113	0.497	0.065	0.820	0.001	0.995	0.060	0.677
HW2	−0.087	0.642	0.205	0.268	0.225	0.509	0.028	0.881	−0.044	0.786
HW3	0.098	0.594	−0.064	0.716	0.452	0.208	0.047	0.791	−0.150	0.337
HW4	0.101	0.209	−0.024	0.758	0.038	0.776	0.093	0.238	−0.065	0.337
Respiratory										
HW1	0.111	0.742	−0.350	0.295	0.784	0.237	0.173	0.608	−0.080	0.791
HW2	0.027	0.940	−0.253	0.471	0.220	0.691	−0.033	0.926	0.435	0.137
HW3	−0.329	0.336	−0.247	0.470	−0.041	0.933	−0.153	0.654	−0.040	0.896
HW4	−0.192	0.189	0.173	0.240	−0.256	0.193	0.193	0.192	**−0.383**	**0.006**

Note: The coefficient with a *p*-value <0.05 is highlighted.

## Supplementary Materials

The following are available online at https://www.mdpi.com/1660-4601/16/22/4568/s1, Figure S1: Mortality rate and median household income by census tract in Shelby County, TN; Table S1: Effects of heatwave days on all-cause mortality on heatwave (HW) days and 1, 2, and 3 lag days; Table S2: Effects of heatwave days on cardiovascular disease mortality on heatwave (HW) days and 1, 2, and 3 lag days; Table S3: Effects of heatwave days on respiratory disease mortality on heatwave (HW) days and 1, 2, and 3 lag days.
