# Supplementary material for: Heatwave Events and Mortality Outcomes in Memphis, Tennessee: Testing Effect Modification by Socioeconomic Status and Urbanicity
Source: Int J Environ Res Public Health. 2019 Nov 18;16(22):4568. doi: 10.3390/ijerph16224568 (PMC6888315; doi:10.3390/ijerph16224568)
Supplement: Supplementary file 1 [file ijerph-16-04568-s001.pdf]

International Journal of
Environmental Research and Public Health

MDPI

Article

Heatwave Events and Mortality Outcomes in Memphis, Tennessee: Testing Effect Modification by Socioeconomic Status and Urbanicity

Ying Li [1], Cem Akkus [2,3], Xinhua Yu [2], Andrew Joyner [4], Jennifer Kmet [5], David Sweat [5], and Chunrong Jia [2,*]

[1] Department of Environmental Health, College of Public Health, East Tennessee State University, Johnson City, TN 37614, U.S.A.; liy005@etsu.edu
[2] School of Public Health, University of Memphis, Memphis, TN 38152, U.S.A.; xyu2@memphis.edu (X.Y.); cjia@memphis.edu (C.J.)
[3] Children's Foundation Research Institute, Le Bonheur Children's Hospital, Memphis, TN 38103, U.S.A.; cem.akkus@lebonheur.org (C.A.)
[4] Department of Geosciences, East Tennessee State University, Johnson City, TN 37614, U.S.A.; joynert@etsu.edu
[5] Shelby County Health Department, Memphis, TN 38105, U.S.A.; jennifer.kmet@shelbycountytn.gov (J.K.); david.sweat@shelbycountytn.gov (D.S.)

* Correspondence: cjia@memphis.edu; Tel.: +1-901-678-2655 (U.S.A.)

Received: date; Accepted: date; Published: date

Supplementary Materials

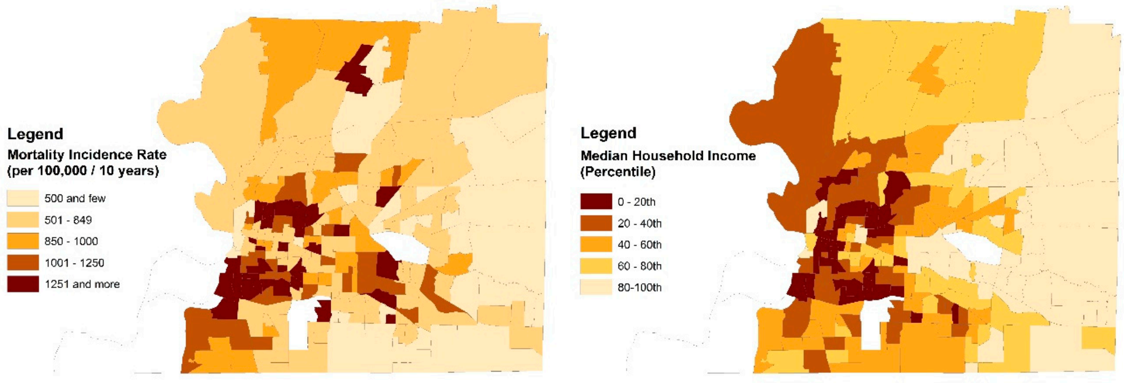

Figure S1. Mortality rate and median household income by census tract in Shelby County, TN.

Table S1. Effects of heatwave days on all-cause mortality on heatwave (HW) days and 1, 2, and 3 lag days.

Table S1A. Heatwave days determined by HW1.

Parameter	Value	HW		lag 1		lag 2		lag 3	
		Estimate	*p* -value	Estimate	*p* -value	Estimate	*p* -value	Estimate	*p* -value
Intercept		–0.1075	0.0001	–0.1069	0.0001	–0.1059	0.0002	–0.1047	0.0002
Year	2009	0.0214	0.2984	0.0212	0.3017	0.021	0.3074	0.0207	0.3129
Year	2010	0.0241	0.2278	0.0233	0.2425	0.0226	0.2571	0.0211	0.2901
Year	2011	0.0684	0.0005	0.0679	0.0006	0.0676	0.0006	0.0668	0.0007
Year	2012	0.0431	0.0304	0.0428	0.0317	0.0426	0.0325	0.042	0.0349
Year	2013	0.0232	0.2381	0.0233	0.2354	0.0236	0.2301	0.024	0.2224
Year	2014	0.0762	<0.0001	0.0762	<0.0001	0.0763	<0.0001	0.0762	<0.0001
Year	2015	0.0868	<0.0001	0.0866	<0.0001	0.0866	<0.0001	0.0863	<0.0001
Year	2016	0.0935	<0.0001	0.0928	<0.0001	0.0924	<0.0001	0.0919	<0.0001
Year	2017	0.1095	<0.0001	0.1097	<0.0001	0.11	<0.0001	0.1104	<0.0001
Year	2008	0	.	0	.	0	.	0	.
DOW	2	–0.0042	0.7836	–0.0038	0.8062	–0.0041	0.7879	–0.0041	0.7926
DOW	3	–0.0158	0.3063	–0.0159	0.3045	–0.0157	0.3095	–0.0156	0.312
DOW	4	0.0091	0.5558	0.0089	0.5639	0.0085	0.5834	0.0085	0.583
DOW	5	–0.0161	0.2991	–0.0157	0.3129	–0.016	0.3025	–0.016	0.3011
DOW	6	0.0227	0.1398	0.0231	0.1328	0.023	0.1335	0.0227	0.1386
DOW	7	0.005	0.7471	0.0054	0.726	0.0054	0.7278	0.0051	0.7395
DOW	1	0	.	0	.	0	.	0	.
Age	80 and plus	0.077	<0.0001	0.077	<0.0001	0.077	<0.0001	0.077	<0.0001
Age	65–79	0	.	0	.	0	.	0	.
Poverty	Low	0.028	0.0012	0.028	0.0012	0.028	0.0012	0.0279	0.0013
Poverty	High	0	.	0	.	0	.	0	.
Urban	Urban	0.3568	<0.0001	0.3567	<0.0001	0.3566	<0.0001	0.3567	<0.0001
Rural	Rural	0	.	0	.	0	.	0	.
Race	White	–0.0319	0.0003	–0.0319	0.0003	–0.0319	0.0003	–0.0319	0.0003
Race	Other	–0.3579	<0.0001	–0.3581	<0.0001	–0.3579	<0.0001	–0.3577	<0.0001
Race	Black	0	.	0	.	0	.	0	.
Sex	Male	0.0761	<0.0001	0.0761	<0.0001	0.0761	<0.0001	0.0761	<0.0001
Sex	Female	0	.	0	.	0	.	0	.
HW	Yes	–0.0396	0.1218	–0.0252	0.3079	–0.013	0.5955	0.008	0.7354
HW	No	0	.	0	.	0	.	0	.
Ozone		0.0001	0.8207	0.0001	0.8622	0.0001	0.9142	0	0.9939
Scale		0.5464		0.5464		0.5464		0.5464	

Table S1B. Heatwave days determined by HW2.

Parameter	Value	HW		lag 1		lag 2		lag 3	
		Estimate	*p*-value	Estimate	*p*-value	Estimate	*p*-value	Estimate	*p*-value
Intercept		–0.1049	0.0002	–0.1042	0.0002	–0.104	0.0002	–0.1045	0.0002
Year	2009	0.0207	0.3125	0.0204	0.3199	0.0203	0.3225	0.0203	0.3223
Year	2010	0.0215	0.2812	0.0207	0.2995	0.0199	0.3195	0.0198	0.3204
Year	2011	0.067	0.0007	0.0665	0.0008	0.0657	0.0009	0.0658	0.0009
Year	2012	0.0423	0.0337	0.0423	0.0336	0.0422	0.0341	0.0421	0.0345
Year	2013	0.0239	0.2249	0.0239	0.2237	0.024	0.2229	0.0239	0.2236
Year	2014	0.0763	<0.0001	0.0763	<0.0001	0.0763	<0.0001	0.0763	<0.0001
Year	2015	0.0864	<0.0001	0.0861	<0.0001	0.0857	<0.0001	0.0856	<0.0001
Year	2016	0.0919	<0.0001	0.0913	<0.0001	0.0909	<0.0001	0.0907	<0.0001
Year	2017	0.1103	<0.0001	0.1103	<0.0001	0.1104	<0.0001	0.1103	<0.0001
Year	2008	0	.	0	.	0	.	0	.
DOW	2	–0.0041	0.7884	–0.0043	0.7803	–0.0041	0.7909	–0.004	0.7942
DOW	3	–0.0157	0.3096	–0.0158	0.3082	–0.0158	0.3066	–0.0155	0.3162
DOW	4	0.0084	0.5852	0.0083	0.5909	0.0082	0.5943	0.0084	0.5844
DOW	5	–0.0161	0.2985	–0.0165	0.289	–0.0164	0.2912	–0.0162	0.2967
DOW	6	0.0228	0.1372	0.0227	0.139	0.0223	0.1473	0.0227	0.1392
DOW	7	0.0053	0.7317	0.0052	0.7341	0.0051	0.74	0.0047	0.759
DOW	1	0	.	0	.	0	.	0	.
Age	80 and plus	0.077	<0.0001	0.077	<0.0001	0.077	<0.0001	0.077	<0.0001
Age	65–79	0	.	0	.	0	.	0	.
Poverty	Low	0.0279	0.0013	0.028	0.0012	0.0279	0.0013	0.028	0.0012
Poverty	High	0	.	0	.	0	.	0	.
Urban	Urban	0.3566	<0.0001	0.3566	<0.0001	0.3567	<0.0001	0.3567	<0.0001
Rural	Rural	0	.	0	.	0	.	0	.
Race	White	–0.0319	0.0003	–0.0319	0.0003	–0.0319	0.0003	–0.0319	0.0003
Race	Other	–0.3578	<0.0001	–0.3578	<0.0001	–0.358	<0.0001	–0.3579	<0.0001
Race	Black	0	.	0	.	0	.	0	.
Sex	Male	0.0761	<0.0001	0.0761	<0.0001	0.0762	<0.0001	0.0761	<0.0001
Sex	Female	0	.	0	.	0	.	0	.
HW	Yes	0.0035	0.9058	0.0161	0.569	0.0292	0.3016	0.0279	0.3091
HW	No	0	.	0	.	0	.	0	.
Ozone		0	0.9744	0	0.9946	0	0.979	0	0.9985
Scale		0.5464		0.5464		0.5464		0.5464	

Table S1C. Heatwave days determined by HW3.

Parameter	Value	HW		lag 1		lag 2		lag 3	
		Estimate	*p*-value	Estimate	*p*-value	Estimate	*p*-value	Estimate	*p*-value
Intercept		–0.1076	0.0001	–0.1054	0.0002	–0.1043	0.0002	–0.1042	0.0002
Year	2009	0.0212	0.3012	0.0209	0.3092	0.0207	0.3132	0.0207	0.3135
Year	2010	0.0238	0.2331	0.0221	0.2683	0.021	0.2943	0.0202	0.3118
Year	2011	0.07	0.0004	0.0676	0.0006	0.0662	0.0009	0.065	0.0011
Year	2012	0.0437	0.0283	0.0425	0.0329	0.0417	0.0365	0.0409	0.0407
Year	2013	0.0235	0.2318	0.0238	0.2263	0.0239	0.2239	0.0239	0.2235
Year	2014	0.0766	<0.0001	0.0763	<0.0001	0.0761	<0.0001	0.0759	<0.0001
Year	2015	0.0862	<0.0001	0.0864	<0.0001	0.0865	<0.0001	0.0865	<0.0001
Year	2016	0.0919	<0.0001	0.0921	<0.0001	0.0922	<0.0001	0.0922	<0.0001
Year	2017	0.1098	<0.0001	0.1102	<0.0001	0.1103	<0.0001	0.1104	<0.0001
Year	2008	0	.	0	.	0	.	0	.
DOW	2	–0.0046	0.7641	–0.0041	0.7887	–0.0042	0.7876	–0.004	0.7931
DOW	3	–0.0166	0.2849	–0.0159	0.3046	–0.0158	0.3082	–0.0158	0.3067
DOW	4	0.0084	0.585	0.0084	0.5882	0.0086	0.5764	0.0085	0.5836
DOW	5	–0.0163	0.2946	–0.0162	0.2976	–0.016	0.3035	–0.0158	0.3094
DOW	6	0.0226	0.1418	0.0228	0.138	0.0228	0.1383	0.0231	0.1324
DOW	7	0.0051	0.7418	0.0052	0.736	0.0053	0.733	0.0052	0.7354
DOW	1	0	.	0	.	0	.	0	.
Age	80 and plus	0.077	<0.0001	0.077	<0.0001	0.077	<0.0001	0.077	<0.0001
Age	65–79	0	.	0	.	0	.	0	.
Poverty	Low	0.0279	0.0013	0.028	0.0013	0.0279	0.0013	0.0279	0.0013
Poverty	High	0	.	0	.	0	.	0	.
Urban	Urban	0.3566	<0.0001	0.3566	<0.0001	0.3566	<0.0001	0.3567	<0.0001
Rural	Rural	0	.	0	.	0	.	0	.
Race	White	–0.032	0.0003	–0.0319	0.0003	–0.0319	0.0003	–0.032	0.0003
Race	Other	–0.3579	<0.0001	–0.3578	<0.0001	–0.3577	<0.0001	–0.3575	<0.0001
Race	Black	0	.	0	.	0	.	0	.
Sex	Male	0.076	<0.0001	0.0761	<0.0001	0.0761	<0.0001	0.0761	<0.0001
Sex	Female	0	.	0	.	0	.	0	.
HW	Yes	–0.0365	0.205	–0.0066	0.8161	0.0107	0.6933	0.0227	0.3932
HW	No	0	.	0	.	0	.	0	.
Ozone		0.0001	0.8041	0	0.939	0	0.9941	0	0.9845
Scale		0.5464		0.5464		0.5464		0.5464	

Table S1D. Heatwave days determined by HW4.

Parameter	Value	HW		lag 1		lag 2		lag 3	
		Estimate	*p*-value	Estimate	*p*-value	Estimate	*p*-value	Estimate	*p*-value
Intercept		–0.1071	0.0001	–0.1075	0.0001	–0.108	0.0001	–0.1072	0.0001
Year	2009	0.0212	0.3021	0.0212	0.3017	0.0211	0.3046	0.021	0.3069
Year	2010	0.0244	0.2249	0.026	0.1941	0.027	0.1779	0.027	0.1775
Year	2011	0.0687	0.0005	0.0698	0.0004	0.0705	0.0004	0.0706	0.0004
Year	2012	0.0433	0.0297	0.0442	0.0266	0.0449	0.0244	0.0449	0.0245
Year	2013	0.0232	0.2377	0.0227	0.2485	0.0224	0.2556	0.0225	0.2528
Year	2014	0.0757	<0.0001	0.0754	0.0001	0.0749	0.0001	0.0749	0.0001
Year	2015	0.087	<0.0001	0.0871	<0.0001	0.0874	<0.0001	0.0873	<0.0001
Year	2016	0.0937	<0.0001	0.0949	<0.0001	0.0948	<0.0001	0.0949	<0.0001
Year	2017	0.1095	<0.0001	0.1091	<0.0001	0.1087	<0.0001	0.1089	<0.0001
Year	2008	0	.	0	.	0	.	0	.
DOW	2	–0.0044	0.7736	–0.0046	0.7671	–0.0034	0.8231	–0.0047	0.7586
DOW	3	–0.0162	0.2955	–0.0169	0.2739	–0.0156	0.3148	–0.0156	0.3131
DOW	4	0.0085	0.5805	0.0077	0.6187	0.0084	0.5854	0.0085	0.5805
DOW	5	–0.0164	0.2904	–0.0164	0.2898	–0.0164	0.2912	–0.017	0.2719
DOW	6	0.0224	0.1449	0.0223	0.1473	0.0237	0.1226	0.0224	0.144
DOW	7	0.0054	0.7274	0.0043	0.7799	0.006	0.6994	0.0058	0.706
DOW	1	0	.	0	.	0	.	0	.
Age	80 and plus	0.077	<0.0001	0.077	<0.0001	0.0769	<0.0001	0.077	<0.0001
Age	65–79	0	.	0	.	0	.	0	.
Poverty	Low	0.0279	0.0013	0.028	0.0012	0.028	0.0012	0.028	0.0012
Poverty	High	0	.	0	.	0	.	0	.
Urban	Urban	0.3566	<0.0001	0.3568	<0.0001	0.3569	<0.0001	0.3567	<0.0001
Rural	Rural	0	.	0	.	0	.	0	.
Race	White	–0.032	0.0003	–0.0319	0.0003	–0.0322	0.0003	–0.0321	0.0003
Race	Other	–0.3582	<0.0001	–0.3588	<0.0001	–0.3583	<0.0001	–0.3584	<0.0001
Race	Black	0	.	0	.	0	.	0	.
Sex	Male	0.0761	<0.0001	0.076	<0.0001	0.0761	<0.0001	0.076	<0.0001
Sex	Female	0	.	0	.	0	.	0	.
HW	Yes	–0.0124	0.3203	–0.0206	0.0994	–0.0255	0.0409	–0.0256	0.0394
HW	No	0	.	0	.	0	.	0	.
Ozone		0.0001	0.7928	0.0002	0.7256	0.0002	0.7109	0.0002	0.7245
Scale		0.5464		0.5464		0.5463		0.5463	

Table S2. Effects of heatwave days on cardiovascular disease mortality on heatwave (HW) days and 1, 2, and 3 lag days.

Table S2A. Heatwave days determined by HW1.

Parameter	Value	HW		lag 1		lag 2		lag 3	
		Estimate	*p*-value	Estimate	*p*-value	Estimate	*p*-value	Estimate	*p*-value
Intercept		–0.0245	0.4043	–0.023	0.4341	–0.0194	0.5086	–0.0227	0.4382
Year	2009	–0.0109	0.6043	–0.0111	0.5953	–0.0121	0.5627	–0.0114	0.5852
Year	2010	0.021	0.3021	0.0199	0.329	0.0153	0.4539	0.018	0.3777
Year	2011	–0.0058	0.7807	–0.0064	0.7577	–0.0082	0.6945	–0.0074	0.7229
Year	2012	0.0079	0.7023	0.0074	0.7219	0.0056	0.7863	0.0064	0.7594
Year	2013	–0.0186	0.3568	–0.0182	0.3656	–0.0179	0.3736	–0.0182	0.3647
Year	2014	–0.0066	0.7437	–0.0066	0.7457	–0.0066	0.7437	–0.0067	0.7398
Year	2015	0.0184	0.3553	0.0182	0.3612	0.0173	0.3856	0.0177	0.3727
Year	2016	0.0167	0.4041	0.0165	0.4092	0.0155	0.4385	0.016	0.4234
Year	2017	0.0252	0.2081	0.0255	0.2014	0.026	0.1935	0.0256	0.2011
Year	2008	0	.	0	.	0	.	0	.
DOW	2	–0.0192	0.2282	–0.0197	0.2178	–0.0193	0.225	–0.019	0.233
DOW	3	–0.0141	0.3789	–0.0139	0.383	–0.0142	0.3743	–0.0136	0.395
DOW	4	–0.0046	0.7719	–0.0051	0.7496	–0.0052	0.7434	–0.0047	0.7679
DOW	5	–0.026	0.1083	–0.0265	0.1024	–0.0263	0.1044	–0.0254	0.1171
DOW	6	0.0144	0.3687	0.014	0.3841	0.0127	0.4287	0.0141	0.3791
DOW	7	–0.0158	0.3244	–0.016	0.3193	–0.0171	0.2852	–0.0169	0.2933
DOW	1	0	.	0	.	0	.	0	.
Age	80 and plus	0.0427	<0.0001	0.0428	<0.0001	0.0425	<0.0001	0.0424	<0.0001
Age	65–79	0	.	0	.	0	.	0	.
Poverty	Low	0.0083	0.3661	0.0082	0.3739	0.0079	0.3917	0.0084	0.3638
Poverty	High	0	.	0	.	0	.	0	.
Urban	Urban	0.1434	<0.0001	0.1434	<0.0001	0.1436	<0.0001	0.1435	<0.0001
Rural	Rural	0	.	0	.	0	.	0	.
Race	White	–0.0111	0.243	–0.0112	0.2379	–0.0115	0.2289	–0.011	0.2467
Race	Other	–0.1421	0.0003	–0.1421	0.0003	–0.1423	0.0003	–0.1415	0.0003
Race	Black	0	.	0	.	0	.	0	.
Sex	Male	0.0366	<0.0001	0.0366	<0.0001	0.0365	<0.0001	0.0364	<0.0001
Sex	Female	0	.	0	.	0	.	0	.
HW	Yes	0.0093	0.7213	0.0246	0.3246	0.067	0.0081	0.042	0.096
HW	No	0	.	0	.	0	.	0	.
Ozone		–0.0002	0.7034	–0.0002	0.641	–0.0003	0.5053	–0.0002	0.6222
Scale		0.3302		0.3302		0.33		0.3301	

Table S2B. Heatwave days determined by HW2.

Parameter	Value	HW		lag 1		lag 2		lag 3	
		Estimate	*p*-value	Estimate	*p*-value	Estimate	*p*-value	Estimate	*p*-value
Intercept		–0.0222	0.4486	–0.0211	0.4719	–0.0224	0.4446	–0.0249	0.3953
Year	2009	–0.0117	0.5774	–0.0122	0.5617	–0.012	0.5668	–0.0109	0.6031
Year	2010	0.0177	0.3853	0.0172	0.399	0.0169	0.4053	0.0209	0.3063
Year	2011	–0.0081	0.699	–0.0089	0.6697	–0.0089	0.6696	–0.0058	0.7796
Year	2012	0.0085	0.6812	0.0083	0.6885	0.0079	0.7047	0.0081	0.697
Year	2013	–0.0184	0.3612	–0.0183	0.3624	–0.0184	0.3598	–0.0187	0.3524
Year	2014	–0.0065	0.7482	–0.0065	0.7477	–0.0066	0.7465	–0.0066	0.7448
Year	2015	0.0171	0.3892	0.0166	0.4045	0.0167	0.4008	0.0183	0.3585
Year	2016	0.014	0.4834	0.0132	0.5083	0.0135	0.4994	0.0165	0.4113
Year	2017	0.0254	0.2039	0.0254	0.2027	0.0254	0.2039	0.025	0.2112
Year	2008	0	.	0	.	0	.	0	.
DOW	2	–0.02	0.2094	–0.0206	0.1973	–0.0191	0.2301	–0.0192	0.2292
DOW	3	–0.0137	0.3895	–0.0145	0.3652	–0.0143	0.3711	–0.0141	0.3781
DOW	4	–0.0057	0.7204	–0.0058	0.7136	–0.0054	0.7323	–0.0045	0.7798
DOW	5	–0.0265	0.1024	–0.0277	0.0873	–0.0259	0.1102	–0.026	0.109
DOW	6	0.0146	0.3631	0.0139	0.3864	0.013	0.4192	0.0144	0.3694
DOW	7	–0.0157	0.3283	–0.0162	0.3108	–0.0167	0.298	–0.0161	0.3171
DOW	1	0	.	0	.	0	.	0	.
Age	80 and plus	0.0427	<0.0001	0.0427	<0.0001	0.0429	<0.0001	0.0427	<0.0001
Age	65–79	0	.	0	.	0	.	0	.
Poverty	Low	0.0081	0.3798	0.0081	0.3771	0.0079	0.3934	0.0084	0.3628
Poverty	High	0	.	0	.	0	.	0	.
Urban	Urban	0.1431	<0.0001	0.1431	<0.0001	0.1434	<0.0001	0.1434	<0.0001
Rural	Rural	0	.	0	.	0	.	0	.
Race	White	–0.0112	0.2406	–0.0112	0.2401	–0.0111	0.2426	–0.0111	0.2448
Race	Other	–0.1409	0.0003	–0.1416	0.0003	–0.1417	0.0003	–0.142	0.0003
Race	Black	0	.	0	.	0	.	0	.
Sex	Male	0.0369	<0.0001	0.0366	<0.0001	0.0366	<0.0001	0.0366	<0.0001
Sex	Female	0	.	0	.	0	.	0	.
HW	Yes	0.0591	0.0426	0.0702	0.0122	0.0724	0.0119	0.0082	0.7764
HW	No	0	.	0	.	0	.	0	.
Ozone		–0.0002	0.6176	–0.0003	0.5947	–0.0002	0.6303	–0.0002	0.7279
Scale		0.3301		0.33		0.33		0.3302	

Table S2C. Heatwave days determined by HW3.

Parameter	Value	HW		lag 1		lag 2		lag 3	
		Estimate	*p* -value	Estimate	*p* -value	Estimate	*p* -value	Estimate	*p* -value
Intercept		–0.0237	0.4194	–0.0203	0.489	–0.0203	0.489	–0.023	0.4317
Year	2009	–0.0109	0.6015	–0.0115	0.5841	–0.0114	0.585	–0.011	0.5978
Year	2010	0.0205	0.3141	0.017	0.4043	0.0159	0.436	0.017	0.4032
Year	2011	–0.0069	0.743	–0.0106	0.6113	–0.0122	0.5622	–0.0118	0.5733
Year	2012	0.0074	0.7206	0.0044	0.8327	0.0039	0.8502	0.0035	0.8674
Year	2013	–0.0186	0.3566	–0.018	0.3711	–0.0181	0.3671	–0.0185	0.3585
Year	2014	–0.0068	0.7367	–0.0072	0.7207	–0.0068	0.736	–0.0071	0.7255
Year	2015	0.0186	0.3499	0.019	0.3405	0.0189	0.3418	0.0187	0.3468
Year	2016	0.017	0.3937	0.0175	0.3822	0.0174	0.3845	0.0171	0.3917
Year	2017	0.0252	0.2077	0.0259	0.1955	0.0258	0.1963	0.0254	0.2043
Year	2008	0	.	0	.	0	.	0	.
DOW	2	–0.0191	0.2316	–0.0192	0.2292	–0.0198	0.2138	–0.0194	0.2223
DOW	3	–0.0138	0.3897	–0.0123	0.4418	–0.0142	0.3737	–0.0146	0.3606
DOW	4	–0.0045	0.7779	–0.0036	0.8219	–0.0041	0.7974	–0.0047	0.7657
DOW	5	–0.026	0.1089	–0.0256	0.1143	–0.0244	0.1324	–0.0246	0.129
DOW	6	0.0145	0.3681	0.0142	0.3748	0.0135	0.4014	0.0149	0.3538
DOW	7	–0.0159	0.3215	–0.0154	0.3358	–0.0167	0.2961	–0.0169	0.2913
DOW	1	0	.	0	.	0	.	0	.
Age	80 and plus	0.0427	<0.0001	0.0429	<0.0001	0.0427	<0.0001	0.0425	<0.0001
Age	65–79	0	.	0	.	0	.	0	.
Poverty	Low	0.0084	0.3606	0.0081	0.3779	0.0082	0.3716	0.0081	0.3791
Poverty	High	0	.	0	.	0	.	0	.
Urban	Urban	0.1432	<0.0001	0.1435	<0.0001	0.1439	<0.0001	0.1441	<0.0001
Rural	Rural	0	.	0	.	0	.	0	.
Race	White	–0.011	0.2468	–0.0113	0.2364	–0.0112	0.238	–0.0112	0.2389
Race	Other	–0.1421	0.0003	–0.1423	0.0003	–0.1424	0.0003	–0.1415	0.0003
Race	Black	0	.	0	.	0	.	0	.
Sex	Male	0.0367	<0.0001	0.0366	<0.0001	0.0364	<0.0001	0.0363	<0.0001
Sex	Female	0	.	0	.	0	.	0	.
HW	Yes	0.0161	0.5713	0.0693	0.0138	0.0747	0.0068	0.065	0.0175
HW	No	0	.	0	.	0	.	0	.
Ozone		–0.0002	0.6715	–0.0003	0.4909	–0.0003	0.503	–0.0002	0.6249
Scale		0.3302		0.33		0.33		0.33	

Table S2D. Heatwave days determined by HW4.

Parameter	Value	HW		lag 1		lag 2		lag 3	
		Estimate	*p*-value	Estimate	*p*-value	Estimate	*p*-value	Estimate	*p*-value
Intercept		−0.0261	0.3758	−0.0281	0.3381	−0.026	0.3752	−0.0257	0.3811
Year	2009	−0.0106	0.6126	−0.0104	0.6184	−0.0106	0.6126	−0.0107	0.6109
Year	2010	0.0225	0.2716	0.0252	0.2179	0.0229	0.2641	0.0226	0.2689
Year	2011	−0.0048	0.8184	−0.003	0.8873	−0.0044	0.8328	−0.0046	0.8278
Year	2012	0.0086	0.6798	0.01	0.6299	0.009	0.6668	0.0089	0.6695
Year	2013	−0.0189	0.347	−0.0198	0.3248	−0.019	0.3451	−0.0189	0.348
Year	2014	−0.0067	0.7404	−0.0073	0.7197	−0.0068	0.7376	−0.0068	0.7383
Year	2015	0.0188	0.3453	0.0193	0.3322	0.0189	0.3419	0.0188	0.3449
Year	2016	0.0176	0.3801	0.0191	0.3407	0.0178	0.3747	0.0177	0.3768
Year	2017	0.0247	0.2161	0.0242	0.2267	0.0247	0.2162	0.0248	0.2141
Year	2008	0	.	0	.	0	.	0	.
DOW	2	−0.0193	0.2261	−0.0193	0.2264	−0.019	0.2342	−0.0194	0.2244
DOW	3	−0.0144	0.3689	−0.0151	0.3447	−0.0141	0.3758	−0.0141	0.3764
DOW	4	−0.0044	0.7833	−0.0045	0.776	−0.0044	0.7829	−0.0044	0.7842
DOW	5	−0.0261	0.108	−0.0262	0.1058	−0.0261	0.1072	−0.0262	0.1062
DOW	6	0.0144	0.3715	0.0145	0.365	0.0148	0.3574	0.0145	0.3677
DOW	7	−0.0158	0.3252	−0.0162	0.3129	−0.0156	0.3306	−0.0157	0.3278
DOW	1	0	.	0	.	0	.	0	.
Age	80 and plus	0.0428	<0.0001	0.0428	<0.0001	0.0427	<0.0001	0.0426	<0.0001
Age	65–79	0	.	0	.	0	.	0	.
Poverty	Low	0.0083	0.365	0.0084	0.361	0.0085	0.3579	0.0084	0.3611
Poverty	High	0	.	0	.	0	.	0	.
Urban	Urban	0.1434	<0.0001	0.1438	<0.0001	0.1434	<0.0001	0.1435	<0.0001
Rural	Rural	0	.	0	.	0	.	0	.
Race	White	−0.0111	0.2427	−0.0111	0.2455	−0.0111	0.2427	−0.0112	0.2394
Race	Other	−0.1423	0.0003	−0.1426	0.0003	−0.1421	0.0003	−0.1421	0.0003
Race	Black	0	.	0	.	0	.	0	.
Sex	Male	0.0366	<0.0001	0.0365	<0.0001	0.0366	<0.0001	0.0367	<0.0001
Sex	Female	0	.	0	.	0	.	0	.
HW	Yes	−0.0048	0.7068	−0.018	0.167	−0.0067	0.6106	−0.0056	0.6629
HW	No	0	.	0	.	0	.	0	.
Ozone		−0.0001	0.8078	0	0.9585	−0.0001	0.8014	−0.0001	0.7857
Scale		0.3302		0.3302		0.3302		0.3302	

Table S3. Effects of heatwave days on respiratory disease mortality on heatwave (HW) days and 1, 2, and 3 lag days.

Table S3A. Heatwave days determined by HW1.

Parameter	Value	HW		lag 1		lag 2		lag 3	
		Estimate	*p*-value	Estimate	*p*-value	Estimate	*p*-value	Estimate	*p*-value
Intercept		0.0017	0.9509	0.0024	0.9292	0.0031	0.9073	0.0034	0.8984
Year	2009	−0.0054	0.7879	−0.0057	0.7732	−0.0058	0.7703	−0.0058	0.7719
Year	2010	−0.0116	0.5243	−0.0129	0.4787	−0.0132	0.4715	−0.0133	0.4666
Year	2011	−0.0262	0.147	−0.0263	0.1464	−0.0266	0.1423	−0.0265	0.1435
Year	2012	−0.0383	0.0502	−0.0387	0.048	−0.0388	0.0478	−0.0384	0.0506
Year	2013	−0.0144	0.4508	−0.0142	0.458	−0.014	0.4654	−0.0139	0.4682
Year	2014	−0.0173	0.345	−0.0171	0.3498	−0.0169	0.355	−0.0168	0.357
Year	2015	0.0049	0.7892	0.0042	0.8179	0.004	0.8269	0.0043	0.8121
Year	2016	−0.0045	0.8042	−0.0052	0.7709	−0.0055	0.7592	−0.0053	0.7667
Year	2017	0.013	0.4813	0.0132	0.4742	0.0134	0.4679	0.0135	0.4649
Year	2008	0	.	0	.	0	.	0	.
DOW	2	0.0053	0.7317	0.0057	0.7113	0.0053	0.7326	0.0053	0.7292
DOW	3	−0.0144	0.3458	−0.0142	0.3515	−0.014	0.3588	−0.014	0.3601
DOW	4	0.0063	0.6875	0.0057	0.7157	0.0053	0.7366	0.0054	0.7282
DOW	5	−0.0029	0.8472	−0.0025	0.8709	−0.0026	0.863	−0.0026	0.8627
DOW	6	−0.0107	0.4824	−0.0095	0.5341	−0.0096	0.5267	−0.0096	0.53
DOW	7	−0.0351	0.0235	−0.0346	0.0257	−0.0345	0.0264	−0.0342	0.0274
DOW	1	0	.	0	.	0	.	0	.
Age	80 and plus	0.0102	0.2184	0.0101	0.2238	0.0101	0.2254	0.01	0.227
Age	65–79	0	.	0	.	0	.	0	.
Poverty	Low	0.0086	0.3275	0.0089	0.3096	0.009	0.3079	0.0089	0.3143
Poverty	High	0	.	0	.	0	.	0	.
Urban	Urban	0.0361	0.0064	0.036	0.0066	0.0358	0.0069	0.0356	0.0072
Rural	Rural	0	.	0	.	0	.	0	.
Race	White	−0.0103	0.2835	−0.0101	0.2939	−0.0101	0.2962	−0.0102	0.2894
Race	Other	−0.0363	0.3834	−0.0385	0.3553	−0.0384	0.3563	−0.0385	0.3555
Race	Black	0	.	0	.	0	.	0	.
Sex	Male	−0.0114	0.1625	−0.0115	0.1617	−0.0115	0.16	−0.0115	0.1602
Sex	Female	0	.	0	.	0	.	0	.
HW	Yes	−0.0416	0.1302	−0.0179	0.4655	−0.0097	0.6904	−0.0097	0.6708
HW	No	0	.	0	.	0	.	0	.
Ozone		0.0006	0.2056	0.0006	0.2359	0.0006	0.2504	0.0006	0.254
Scale		0.1641		0.1642		0.1642		0.1642	

Table S3B. Heatwave days determined by HW2.

Parameter	Value	HW		lag 1		lag 2		lag 3	
		Estimate	*p*-value	Estimate	*p*-value	Estimate	*p*-value	Estimate	*p*-value
Intercept		0.0016	0.9514	0.0029	0.9159	0.0037	0.8899	0.0037	0.8899
Year	2009	−0.0052	0.7936	−0.0056	0.7782	−0.0058	0.7699	−0.0058	0.7701
Year	2010	−0.0111	0.5428	−0.0129	0.4792	−0.014	0.4443	−0.0139	0.4457
Year	2011	−0.0258	0.1543	−0.0263	0.1475	−0.0272	0.1352	−0.0271	0.1357
Year	2012	−0.0395	0.044	−0.039	0.0468	−0.0389	0.047	−0.0389	0.0472
Year	2013	−0.0141	0.46	−0.0139	0.4683	−0.0138	0.4717	−0.0138	0.4717
Year	2014	−0.017	0.3532	−0.017	0.3533	−0.017	0.354	−0.017	0.354
Year	2015	0.0052	0.7734	0.0044	0.8099	0.0041	0.8231	0.0041	0.8223
Year	2016	−0.0036	0.8422	−0.0048	0.7902	−0.0057	0.7529	−0.0056	0.7559
Year	2017	0.0133	0.4716	0.0135	0.4641	0.0136	0.461	0.0136	0.461
Year	2008	0	.	0	.	0	.	0	.
DOW	2	0.0057	0.7113	0.0055	0.7221	0.0052	0.7362	0.0052	0.736
DOW	3	−0.015	0.3277	−0.0141	0.357	−0.0141	0.356	−0.0141	0.3561
DOW	4	0.0056	0.7202	0.0054	0.7301	0.0053	0.7335	0.0053	0.7335
DOW	5	−0.0028	0.8552	−0.0025	0.8705	−0.0026	0.8633	−0.0026	0.863
DOW	6	−0.0106	0.4863	−0.0095	0.5322	−0.0098	0.5194	−0.0098	0.52
DOW	7	−0.0353	0.0227	−0.0346	0.0256	−0.0345	0.026	−0.0345	0.0263
DOW	1	0	.	0	.	0	.	0	.
Age	80 and plus	0.0103	0.215	0.01	0.227	0.01	0.2268	0.01	0.2266
Age	65–79	0	.	0	.	0	.	0	.
Poverty	Low	0.009	0.3069	0.009	0.3083	0.009	0.3045	0.009	0.3048
Poverty	High	0	.	0	.	0	.	0	.
Urban	Urban	0.0358	0.0069	0.036	0.0067	0.0357	0.007	0.0357	0.0071
Rural	Rural	0	.	0	.	0	.	0	.
Race	White	−0.0096	0.3199	−0.0099	0.3025	−0.01	0.2988	−0.01	0.2988
Race	Other	−0.0389	0.3502	−0.0385	0.3558	−0.0382	0.3585	−0.0383	0.3582
Race	Black	0	.	0	.	0	.	0	.
Sex	Male	−0.0116	0.1573	−0.0116	0.1586	−0.0114	0.1641	−0.0114	0.1634
Sex	Female	0	.	0	.	0	.	0	.
HW	Yes	−0.0446	0.1244	−0.0146	0.5736	0.0006	0.9842	−0.0006	0.9814
HW	No	0	.	0	.	0	.	0	.
Ozone		0.0006	0.2102	0.0006	0.2493	0.0005	0.2675	0.0005	0.2662
Scale		0.1641		0.1642		0.1642		0.1642	

Table S3C. Heatwave days determined by HW3.

Parameter	Value	HW		lag 1		lag 2		lag 3	
		Estimate	*p*-value	Estimate	*p*-value	Estimate	*p*-value	Estimate	*p*-value
Intercept		0.0016	0.9515	0.0018	0.9461	0.0022	0.934	0.0024	0.9291
Year	2009	−0.0053	0.7891	−0.0055	0.7845	−0.0054	0.785	−0.0055	0.7811
Year	2010	−0.0124	0.4968	−0.012	0.513	−0.0121	0.509	−0.0121	0.5059
Year	2011	−0.0246	0.1764	−0.0256	0.1586	−0.0243	0.1837	−0.024	0.1902
Year	2012	−0.038	0.0528	−0.0381	0.0522	−0.0379	0.0534	−0.0373	0.0577
Year	2013	−0.0141	0.4593	−0.014	0.4636	−0.0139	0.468	−0.0139	0.4671
Year	2014	−0.017	0.3522	−0.0167	0.36	−0.0164	0.3686	−0.0165	0.3677
Year	2015	0.0039	0.8298	0.004	0.8256	0.0041	0.8221	0.004	0.8244
Year	2016	−0.0058	0.7471	−0.0057	0.7505	−0.0056	0.7551	−0.0057	0.7525
Year	2017	0.0132	0.4737	0.0134	0.4684	0.0134	0.4655	0.0134	0.4663
Year	2008	0	.	0	.	0	.	0	.
DOW	2	0.005	0.7458	0.0055	0.7197	0.0053	0.729	0.0055	0.7197
DOW	3	−0.0146	0.3399	−0.0146	0.3405	−0.0137	0.3709	−0.0136	0.3738
DOW	4	0.0057	0.7163	0.005	0.7493	0.0048	0.7573	0.0061	0.6983
DOW	5	−0.0027	0.8598	−0.0028	0.8561	−0.0033	0.8269	−0.0031	0.8382
DOW	6	−0.01	0.5119	−0.0102	0.5051	−0.0097	0.5253	−0.0096	0.5279
DOW	7	−0.0347	0.0253	−0.0347	0.0251	−0.0347	0.0254	−0.0343	0.0272
DOW	1	0	.	0	.	0	.	0	.
Age	80 and plus	0.0099	0.2355	0.0099	0.233	0.0101	0.224	0.0102	0.2176
Age	65–79	0	.	0	.	0	.	0	.
Poverty	Low	0.0089	0.3142	0.0088	0.3155	0.0087	0.3243	0.0087	0.3208
Poverty	High	0	.	0	.	0	.	0	.
Urban	Urban	0.0356	0.0072	0.0359	0.0068	0.0358	0.0069	0.0355	0.0074
Rural	Rural	0	.	0	.	0	.	0	.
Race	White	−0.0102	0.2911	−0.0102	0.2911	−0.0102	0.2907	−0.0101	0.2931
Race	Other	−0.0383	0.3572	−0.0385	0.3548	−0.0386	0.3538	−0.0385	0.3545
Race	Black	0	.	0	.	0	.	0	.
Sex	Male	−0.0116	0.1569	−0.0113	0.1686	−0.0115	0.1587	−0.0115	0.159
Sex	Female	0	.	0	.	0	.	0	.
HW	Yes	−0.0303	0.2954	−0.0307	0.3251	−0.0265	0.3101	−0.0261	0.2839
HW	No	0	.	0	.	0	.	0	.
Ozone		0.0006	0.2031	0.0006	0.2163	0.0006	0.223	0.0006	0.2283
Scale		0.1642		0.1642		0.1642		0.1642	

Table S3D. Heatwave days determined by HW4.

Parameter	Value	HW		lag 1		lag 2		lag 3	
		Estimate	*p*-value	Estimate	*p*-value	Estimate	*p*-value	Estimate	*p*-value
Intercept		0.0017	0.9486	0.0022	0.9357	0.0032	0.9052	0.0037	0.8917
Year	2009	–0.0053	0.789	–0.0056	0.7779	–0.0059	0.7675	–0.0059	0.7668
Year	2010	–0.0104	0.5725	–0.0092	0.6164	–0.0085	0.6428	–0.0148	0.4232
Year	2011	–0.0251	0.1671	–0.0234	0.1971	–0.0241	0.1843	–0.0276	0.1292
Year	2012	–0.0366	0.0622	–0.0362	0.0653	–0.0355	0.0711	–0.0393	0.0455
Year	2013	–0.0146	0.4445	–0.0145	0.4482	–0.0156	0.4156	–0.0135	0.4792
Year	2014	–0.0178	0.3297	–0.0184	0.3152	–0.0185	0.3114	–0.0168	0.359
Year	2015	0.0054	0.7675	0.005	0.7829	0.0045	0.8061	0.0039	0.8283
Year	2016	–0.0037	0.8361	–0.0024	0.8955	–0.0042	0.814	–0.006	0.7409
Year	2017	0.0124	0.5005	0.0119	0.5172	0.0117	0.5267	0.0138	0.4535
Year	2008	0	.	0	.	0	.	0	.
DOW	2	0.005	0.7461	0.0051	0.7408	0.0055	0.7204	0.0051	0.7411
DOW	3	–0.0141	0.3568	–0.0147	0.3371	–0.0142	0.3543	–0.0141	0.3557
DOW	4	0.006	0.7006	0.0048	0.7585	0.0051	0.7441	0.0053	0.7356
DOW	5	–0.003	0.8451	–0.0032	0.835	–0.0039	0.7983	–0.0026	0.8664
DOW	6	–0.0105	0.4895	–0.0108	0.4765	–0.0098	0.5193	–0.0097	0.5233
DOW	7	–0.0339	0.0288	–0.0359	0.0206	–0.0353	0.023	–0.0346	0.0256
DOW	1	0	.	0	.	0	.	0	.
Age	80 and plus	0.0096	0.2462	0.0097	0.2425	0.0091	0.2754	0.0101	0.2257
Age	65–79	0	.	0	.	0	.	0	.
Poverty	Low	0.0091	0.2993	0.009	0.3066	0.0084	0.3421	0.0092	0.2966
Poverty	High	0	.	0	.	0	.	0	.
Urban	Urban	0.0351	0.008	0.035	0.0082	0.0355	0.0073	0.0358	0.0069
Rural	Rural	0	.	0	.	0	.	0	.
Race	White	–0.0109	0.2592	–0.011	0.2549	–0.0115	0.2325	–0.0098	0.3102
Race	Other	–0.0359	0.3891	–0.0382	0.3578	–0.0396	0.3411	–0.0381	0.3607
Race	Black	0	.	0	.	0	.	0	.
Sex	Male	–0.0112	0.1713	–0.0108	0.1863	–0.011	0.1782	–0.0114	0.1657
Sex	Female	0	.	0	.	0	.	0	.
HW	Yes	–0.0176	0.1626	–0.0251	0.0443	–0.021	0.0844	0.0033	0.7882
HW	No	0	.	0	.	0	.	0	.
Ozone		0.0007	0.1755	0.0007	0.1575	0.0007	0.1702	0.0005	0.2847
Scale		0.1641		0.164		0.1641		0.1642	
